# Determinants of antenatal care dropout among mothers who gave birth in the last six months in BAHIR Dar ZURIA WOREDA community; mixed designs

**DOI:** 10.1186/s12913-020-05674-9

**Published:** 2020-09-10

**Authors:** Yibeltal Alemu Bekele, Tadesse Ejigu Tafere, Amanu Aragaw Emiru, Henok Biresaw Netsere

**Affiliations:** 1 Department of Reproductive Health and Population Studies, School of Public Health, Bahir Dar University, Bahir Dar, Ethiopia; 2 Department of Nursing, School of Health science, Bahir Dar University, Bahir Dar, Ethiopia

**Keywords:** Antenatal care, ANC visit, Dropout, Determinant, Maternal health, Ethiopia

## Abstract

**Background:**

Antenatal care is the care provides for a pregnant mother to improve the health of the mother and her baby. But in the World including Ethiopia still, mothers do not receive the required number of antenatal care visits. Therefore, the main aim of this study was to identify determinants of Antenatal care visit dropout in Bahir Dar Zuria Woreda North West Ethiopia.

**Methods:**

The study was community-based unmatched case-control study that employed both quantitative and qualitative data. For the quantitative part, 134 cases and 266 controls (total 400) women who gave births in the last six months prior to the study in Bahir Dar Zuria Woreda were enrolled. Data were collected through face to face interviews from March 1 to 30, 2018 using a structured questionnaire. Bivariate and multivariate analysis was used. 95% confidence interval and *P*-value was used to measure the level of significance. For the qualitative part, six FGDs were conducted and open code software was used for the analysis of the data. The finding was narrated by triangulating with the quantitative findings.

**Result:**

Being far distance (AOR 7.26; 95% CI 4.23, 23.01), not having a companion (AOR 3.49; 95% CI; 2.39, 8.44), lack of knowledge (AOR 2.57; 95% CI; 1.25, 5.28), poor wealth index (AOR; 3.36, 95% CI 1.71, 6.62) and not developing a danger sign (AOR 2.18; 95% CI 2.28, 7.64) were predictors of ANC dropout. In addition to this, in the qualitative finding, the socio-culture of the community, attitudes, experience, and perception of the existing services and service provisions were also determinants of ANC drop out.

**Conclusion:**

Socio-cultural, economic, accessibility, and individual factors were determinants of ANC visit drop out. In addition, the behavior of the professional, the mother understands of the existing services, and their perception about ANC influenced ANC dropout.

## Plain English summary

Plain English summary Antenatal care is the care provided for the mother during pregnancy to improve the health of the mother and unborn baby. World health organization recently recommended a minimum of 8 contacts but according to our country Ethiopia ministry health, still, practice-focused antenatal natal care (FANC) recommend a minimum four visits. This study was trying to answer the determinants of antenatal care visit drop out through structured interview questionnaires and explore the perception, attitude, and experience of the respondents through an in-depth interview and focused group discussion.

The participants were asked their socio-demographic characteristics, about Antenatal care services utilization, behavior, attitude and the challenges they face during antenatal care services utilization.

There were 400 participants in the study. This study shows that on average women start antenatal care at 16 weeks and 5 days. Short distances from the facility, knowledge on the timing of ANC, wealth index of the family, a companion person, developed danger sign and their perception attitude and experience of the services were the determinants of antenatal care visit dropout.

In conclusion, Cultural and economic determinants, health services related, accessibility and reproductive history were determinants of ANC drop out. In addition, the behavior of the professional, the mothers understanding of the existing services and their perception of the mother of ANC have influenced ANC dropout. Policy action will be required to farther improve ANC service utilization.

## Background

Globally around 303,000 maternal deaths, 2.7 newborn deaths, and 2.6 million stillbirths occur every year. From this 99%, of all deaths occur in developing countries. Sub-Saharan Africa accounts for more than half of the global maternal mortality and morbidity [1, 2]. Many maternal and prenatal deaths occur in women who have received inadequate and no utilization of ANC. Many of these opportunities continue to be missed in Africa, even though over two-thirds of pregnant women receive at least one antenatal visit [3]. Nevertheless, true progress has been made globally in terms of increasing access and use; still, there were pregnant mothers’ in the world drop out from the initiating ANC services [3].

Globally 85% of pregnant women access antenatal care at least once but around 21% of pregnant women from all pregnancy in the global was a dropout from the required ANC visit [4]. Currently, WHO develops a global strategy for women’s, children’s, and adolescent health with the aims of ending all preventable causes of maternal, child, and adolescent death and maintaining the health and wellbeing’s in the era of sustainable development goals (SDG). To achieve the goal every pregnant woman and the baby need to receive the quality of care throughout her pregnancy, childbirth, and the postnatal period [5]. For this WHO issued a new global guideline on routine ANC recommending a minimum of eight ANC contacts [6]. Although, the model is not put into practice in Ethiopia rather the country is still practicing the focused antenatal care model, a model with a minimum of four ANC visits [7, 8].

The problem of antenatal care visit dropout was more severe in developing country. Sub-Sahara region was one of the African regions having a high ANC dropout rate. In the region around 30% of pregnant mothers among all births in the region was a dropout from the services [4, 9]. According to a report on demographic health survey conducted in Sub-Sahara countries shows that the proportion of mothers, who drop out of the antenatal care services ranges from 37% to 55% [10, 11].

Ethiopia is one of the Sub-Saharan countries having high maternal and newborn mortality according to the 2016 Ethiopian Demographic Health Survey (EDHS) report shows that around 412 women deaths were recorded per 100,000 live births [12]. Although declining aternal death in Ethiopia still, it was high when comparing to the global prevalence. According to EDHS 2016 report, 30% of pregnant mothers in the country drop out the required numbers of antenatal care visits [12].

Different literature showed that women who received a high quality of ANC, age of the mother, testing for HIV, distances from the health facilities, family size, family Wealth index, mode of transportation to seek care, knowledge on pregnancy-related problems, knowledge about antenatal care service directly affects the adequate utilization of antenatal care services utilization [13–16]. In addition, qualitative findings also show that knowledge on ANC services and complication, attitude and perceptions of the existed ANC services were influence the utilization of the services [17].

A different cross-sectional study conducted in Ethiopia also shows that receiving a high quality of ANC care, living with a short distance from the health facility, women involving in the household decision making, having a small family size, having good knowledge with regard to ANC service, positive attitude towards ANC services were determinates of four or more antenatal care services utilization [18–20].

In Ethiopia, the minatory of health recommended pregnant women have at least four ANC visits. However, significant numbers of pregnant women drop out before they received the numbers of ANC Visit. Moreover, the services are not maintaining the women they initiate antenatal care services until they completed the recommended level. Currently, the government of Ethiopia is committed to achieving the Sustainable Development Goal 3 (SDG-3), through improving the maternal health care services utilization [12, 21].

It’s recognized that adequate utilization of the recommended antenatal care visits is important to improve both the health of the mother and the unborn baby. However, there is limited finding on why women fail to use the recommended subsequent antenatal care visits in Ethiopia especially in the study area. As a result, a context-specific study is important since the country have diversified population based on (ethnicity, culture, belief and religious). Therefore the aim of this study was to identify the determinants of antenatal care visit dropout at the community level.

## Methods and materials

### Study area

The study was conducted at Bahir Dar Zuria Woreda; it is located around Bahir Dar city administration 564 km far from capital Addis Ababa. The Woreda consists of 9 clusters. All the clusters in the woreda were rural. The total population of the Woreda was an estimated 220,410 out of which around 48.6% were female. The number of mothers who gave birth in the Woreda in the last 6 months from August first to January last 2018 was 2599 from that 86.4% have a history of ANC and 813 mothers did not complete the required ANC services. In the Woreda, there are 9 health centers. The total number of health professionals in the Woreda is 224; in addition, there are 32 health extension workers [22].

### Study design and study period

A community-based unmatched case-control study employed both quantitative and qualitative data collection. The study was conducted from March 1 to 30, 2018. Women who have a history of ANC but the outcomes of birth were abortion or stillbirths were not included in this study.

### Sample size determination

The sample size was determined by considering the main factor for ANC dropout based on previous literature by using Epi- info version 7.1. Based on research conducted in Nepal, (since there is limited literature conducted in Ethiopia and Africa) [23]. On the assumption of power 90%, confidences interval 95%, control to case ratio 2, proportions of control exposed 35.57%, and proportions of the case exposed 28.86% with 10% non-response rate, the final sample size was 134 cases and 266 controls (a total of 400 participants).

### Sampling procedures

All nine Kebeles (clusters) in the study area were included. The study participants were selected based on the outcome variable of ANC visit dropout and ANC visit completion. The health extension worker’s registration book (family folder) was used as a frame to identify mothers who gave birth for the last 6 months prior to the study period. Then, home to home visit was conducted to ascertain whether the mothers had completed the required visit or not.

Subsequently, all the cases, which dropped out from the antenatal care services, were selected by a simple random sampling technique using the list of mothers as a sampling frame. On the other hand, all mothers who receive the required numbers of antenatal care visits -attending four or more ANC visits- were categorized as control groups. The controls were also identified by using a simple random sampling method.

For the qualitative part, the participants were selected purposively from the study area, through the collaboration of the kebele leaders, health extension workers, and women health development army. The participants were the expectant mothers (who did not participate in the quantitative part) and husbands.

### Data collection tools and procedures

The quantitative parts of the data were collected using a questionnaire. The questionnaire was adopted and modified from the Ethiopian DHS and other previous studies. It was translated into Amharic and then translated back to English for consistency.

The data collectors for the survey part were 9 people who living in the cluster and had completed 10th grade. Four supervisors who hold a Nurse degree were also included in the data collection. The data collectors speak and write the local language and they have experience in data collection. The data collector received 2 days of training before data collection. The training was given in Amharic on how to ask and fill the question, selection criteria of the mother, and how to approach the mothers. Before the actual data collection, the questionnaire was pre-tested for completeness and appropriateness to the local context on 10% (40) mothers.

For the qualitative part, a total of 6 FGD and 4 key informants’ interviews were conducted. The participants were those mothers who have a history of ANC, mothers with no history of ANC, prim-gravid mothers and multi-gravid mothers. Each group of the FGD consists of six people. The duration of the FGD was between discussion was taken 50 min to 1 h, 15 min. Two persons were assigned for note-taking and tape recording while the principal investigator facilitated the discussion.

### Data processing and analysis

Data were cleaned, coded, and entered into Epi-info version 7.1 then exported to SPSS version 23 for analysis. A descriptive analysis was carried out to see the distribution of independent variables. Binary logistic regression was used to examine associations between the dependent variable and each independent variable. Based on the bivariate analysis those factors whose crude associations to antenatal care dropout at *p* < 0.2 were entered into the multivariable analysis to get adjusted odds ratio.

The strength of association was determined by using the crude odds ratio in the binary logistic regressions and adjusted odds ratio in multivariable logistic regression analysis. *P*-values and 95% confidence interval were used to determine the level of significance of the association. *P* < 0.05 considered as statistically significant. Hosmer and Lemeshow Test were used for checking the model fitness of logistic regressions.

Data were collected from a sample quantitative and qualitative one. For the qualitative part, the data were analyzed using open code software with the protocol numbers of. The collected data were transcribed word-by-word into plain text and translated into English. After developing codes, all the issues discussed under those codes were identified as a theme. Finally, the identified themes were arranged into coherent groupings and narrated the finding in descriptive ways in convergent with the quantitative finding for the report.

## Result

### Socio-demographic characteristic

A total of 400 mothers participated in this study with 100% response rate. Forty-two (31.4%) and eighty-six (32.3%) of the respondents among cases and controls respectively were in the age group of 25-29 years. The mean age of the respondents was 28.47 years (SD ± 5.47). One hundred twenty-seven (94.8%) and two hundred sixty-three (98.8%) of the respondents among cases and controls respectively were married. See Table 1 for more details.

**Table 1:** Socio-demographic characteristics of the respondents in Bahir Dar Zuria Woreda North West Ethiopia, 2018

Variable	ANC dropout	
	Case (< 4 visits)	Controls(≥ 4 visits)
Age		
< 20 years	3 (2.2%)	6 (2.3%)
20–24 years	16 (14.9)	73 (27.4%)
25–29 years	42 (31.4%)	86 (32.3%)
30–34 years	47 (35%)	66 (24.8%)
≥35 years	26 (19.4%)	35 (13.2%)
Educational status of the mother		
Not attained formal education	120 (89.5%)	230 (86.5%)
Primary level (Grade 1to 8)	12 (8.9%)	29 (10.9%)
Secondary (Grade 9 to 12)	2 (1.5%)	7 (2.6%)
Marital status		
Married	127 (94.8%)	263 (98.8%)
Not married	7 (5.2%)	3 (1.2%)
Occupation of the mother		
Farmer	130 (97%)	254 (95.5%)
Other*	4 (3%)	12 (4.5%)
Husband educational status		
Not attained formal education	116 (91.3%)	214 (81.4%)
Attended formal education	11 (8.7%)	49 (18.6%)
Husband occupation		
Farmer	118 (92.9%)	224 (85.2%)
Other**	9 (7.1%)	39 (14.8%)

* housewive, student and dialy laborer

** driver, daily laborer and governmental employee

### Cultural and economic characteristic

Sixty (44.8%) and eighty-one (30.5%) of the respondents among cases and controls respectively had poor wealth index respectively. Sixty-three (47%) and 204(76.7%) of the respondents among cases and controls respectively have accompanied person during her ANC visits. Eighty-eight (65.6%) and 234(87.9%) of the respondent among cases and controls respectively were knowledgeable about antenatal care. The qualitative finding also showed that the sources of information were health professionals, women health development army, health professionals, health extension workers and friends. Thirty-eight (28.6%) and 149(87.9%) of the respondents among cases and controls had an unfavorable attitude towards antenatal care service respectively (Cronbach alpha score 0.83). Ninety-four (70%) of respondents among cases and 187(70.3%) of respondents among controls had both husband and wife as the principal income generator for the household. See Table 2 for more details.

**Table 2:** Socio-cultural and economic characteristics of the respondents in Bahir Dar Zuria Woreda Northwest Ethiopia 2018

Variable	ANC dropout	
	Case (< 4 visits)	Controls(≥ 4 visits)
**Religion**		
Orthodox	134 (100%)	260 (97.7%)
Muslim	0	6 (2.3%)
**Wealth index**		
Poor	60 (44.8%)	81 (30.5%)
Middle	30 (22.4%)	60 (22.5%)
Rich	44 (32.8%)	125 (46.9%)
**Accompanied during ANC visit**		
Yes	63 (47%)	204 (76.7%)
No	71 (53%)	62 (23.3%)
**Who goes with you**		
Husband	50 (79.3%)	169 (82.8%)
child	5 (7.9%)	19 (9.3%)
respondent mother /Husbands mother	8 (12.8%)	16 (7.9%)
**Knowledge of the mother on ANC**		
Knowledgeable	88 (65.6%)	234 (87.9%)
Not knowledgeable	46 (34.4%)	32 (12.1%)
**Attitude toward ANC**		
Favorable	38 (28.4%)	149 (56%)
Not favorable	96 (71.6%)	117 (44%)
**Principal Income generator**		
Husband	33 (24.6%)	70 (26.3%)
Wife	1 (0.7%)	8 (3%)
Both	94 (70%)	187 (70.3%)
My parents	6 (4.7%)	1 (.4%)
**Family size**		
1–3	19 (14.2%)	70 (26.3%)
4–5	48 (35.8%)	94 (35.3%)
Above 5	67 (50%)	102 (38.4%)

### Reproductive and the health services related characteristics

Seventy-seven (57.5%) of respondents among cases and 136 (51.1%) of respondents among controls were multi-gravid. Eighty-six (64.2%) of respondents among cases and 139 (52.3%) of the respondents among controls were multi-para. The average weeks of starting ANC follow up among cases were 17 weeks and 1 days, and among controls were 12 weeks 4 days. One hundred sixteen (86.6%) of the respondents among cases and two hundred forty-two (90.9%) of the respondents among controls have wanted pregnancy. One hundred thirty (97%) of the respondents among cases and two hundred thirty-seven (89%) of the respondents among controls traveled to the health center on foot. See Table 3 for more detail.

**Table 3:** Reproductive health characteristics of the respondents in Bahir Dar Zuria Woreda North West Ethiopia, 2018(*n* = 400)

Variable	ANC dropout	
	Case (< 4 visits)	Controls(≥ 4 visits)
**Number of pregnancy**		
Gravid-1	20 (14.9%)	60 (22.5%)
Multi-gravid	77 (57.5%)	136 (51.1%)
Grand multi gravid	37 (27.6%)	70 (26.4%)
**Parity**		
Para-I	22 ((16.4%)	69 (25.9%)
Multi-para	86 (64.2%)	139 (52.3%)
Grand-multi para	26 (19.4%)	58 (21.8%)
**Wanted pregnancy**		
Yes	116 (86.6%)	242 (90.9%)
No	18 (13.4%)	24 (9.1%)
**Distance**		
Less than 30 min	14 (10.4%)	100 (37.6%)
30 min to one hour	46 (34.3%)	100 (37.6%)
More than one hour	74 (55.3%)	66 (24.8%)
**Mode of transportation**		
On foot	130 (97%)	237 (89%)
By car	4 (3%)	29 (11%)

### Danger sign during pregnancy

**Fig. 1:**
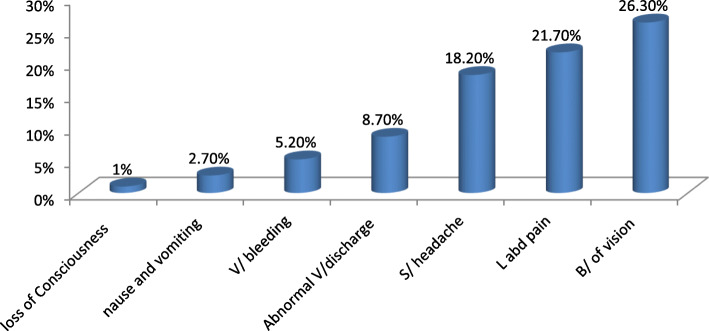
Danger sign during pregnancy among ANC attendant mothers in Bahir Dar Zuria Woreda North West Ethiopia 2018. It shows the numbers of pregnant women who develop a danger sign during her last pregnancy

Forty-four (30.6%) of the respondents among cases and 161(60.5%) of the respondents among controls developed at least one danger sign during their pregnancy time. One hundred five (26.3%), eighty-seven (21.7%), and seventy-five (18.2%) of respondents among all were developed blurring of vision, lower abdominal pain, severe headache. See Fig. 1 for more details.

### Determinants of ANC dropout

In bivariate analysis family size, parity, gravidity, attitude, educational status, knowledge about ANC, distance from the health facility, accompanying person, wealth index, and developed danger signs during pregnancy, were found a candidate variable for multivariate analysis at *P* < 0.2. On multivariate analysis distance from the health facility, wealth index, knowledge of ANC, accompanying person, and developed danger signs during pregnancy were statistically significant at *P*-value < 0.05.

The odds of ANC drop out among women who travel more than 1 h to reach the health facility was 7.26 times higher than those women who traveled less than 30 min (AOR = 7.26; 95% CI; 4, 23, 23.01) and the odds of ANC drop out among women who traveled thirty to 1 h to reach the health facility was 3.12 times higher than those women who traveled less than 30 min (AOR = 3.12; 95% CI; 2.55, 8.69). See Table 4 for more details.

**Table 4:** Factors associated with ANC dropout in Bahir Dar Zuria Woreda North West Ethiopia 2018 (*n* = 400)

Variable	COR(95%,CI)	AOR(95%,CI)
**Family size**		
< 5	1	1
≥ 5	2.12 (1.37,3.32)	1.82 (0.79, 4.12)
**Gravidity**		
Gravid-I	1	1
Multi-gravid	1.69 (0.83,3.02)	0.788 (0.05,10.69)
Grand multi-gravid	1.59 (0.95,3.03)	0.46 (0.043,4.81)
**Distance**		
< =30 min	1	1
30 to 60 min	3.28 (2.41,5.8)	3.124 (2.55,8.69)**
More than one hour	8.0 (4.18,15.35)	7.26 (4,23,23.01)***
**Person go with you**		
Yes	1	1
No	3.71 (2.38,5.77)	3.49 (2.39,8.44)***
**Educational status**		
Attained formal Education	1	1
Not attained formal Educated	1.34 (1.85,4.166)	1.89 (0.15,1.05)
**Knowledge**		
Knowledgeable	1	1
Not knowledgeable	3.82 (2.29,6.39)	2.57 (1.25,5.28)*
**Wealth index**		
Rich	1	1
Middle	1.42 (0.85,2.57)	2.024 (0.93, 4.4)
poor	2.10 (1.35,3.53)	3.36 (1.71, 6.62)***
**Danger signs**		
Yes	1	1
No	3.47 (2.24,5.41)	3.18 (2.28,7.64)***
**Attitude**		
Favorable	1	1
Non favorable	3.22 (2.22,5.03)	1.72 (0.87,3.39)

*p-value < 0.05;

**p-value < 0.01;

***p-value < 0.001

Similarly, the qualitative finding also explains, distance from the facility was not an impediment to ANC follow up.

34 years old female FGD participant said, “*...In our neighborhood, the health center is too far. There is no nearby road and means of transportation to reach the health center. We travel one way two to three hours, because of that, we are facing a problem …*”.

39 years old male FGD participant said, “*… we built health post here in our district (kebele) but still the government not assigned health extension workers...”*

The odds of ANC drop out among women who had from poor family wealth index were 3.36 times higher than those women who had a rich wealth index (AOR = 3.36 95% CI; 1.71, 6.62). See Table 4 for more details.

Most of the FGD participants specify the importance of utilizing the services but they frequently state the indirect costs of ANC. Like transportation costs, loss from agricultural and household activity constrains them for not utilization services.

37 years old male FGD participant said “*...When we are going to the health center, we pay around 50 Ethiopian Birr’s for the motorcycle to reach the health center and turn back home for a single person...”*

24 years old female FGD participant said, “*… Even if he [husband] told me to go there [health facility] I did not do that because he can’t cover all the agricultural activity …*”.

The odds of ANC drop out among mothers who had poor knowledge of ANC services were 2.57 times higher than those mothers who had knowledgeable (AOR = 2.57; 95% CI;1.25,5.28). See Table 4 for more details.

This finding was supported by qualitative data. Knowledge of antenatal care services was overall high among all the participants of FGD including men. But few of the respondents were lack awareness of the existed ANC services (the importance, adequacy of the services, and frequency of the services).

35 years old female FGD participant said *“. Antenatal care is a service that I was attained to take TT (Mengaga Kolf) vaccine and iron tablet that is vital for mental developments of the baby …*.”

35 years old male FGD participant said, “*…*. P*regnancy follow up is a governmental principle, that mother is going to the health center for examination to execute the policy …*”.

The odds of ANC drop out among women who had no accompanied person with her on each visit was 3.49 times higher than those women who had to accompany person (AOR = 3.49 95%; CI = 2.39, 8.44). See Table 4 for more details.

This finding was in line with the qualitative data. The participants were explained that, the importance of involved the accompanying person on each ANC visit. But they said the attitude of the health professional, the workload, and lack awareness on the existed services were the determinants of not involving the accompanying person.

37 years old male FGD participant said, “*When I go with her, first I need to check and know our HIV status. We all are at risk of getting the virus because it transmits through a needle and other sharp materials so we need to examine for HIV both at a time but they ignored us. They examine our wife cheating (teserka) from us”.*

The odds of ANC drop out among women who had not to develop danger signs were 3.18 times higher than those women who develop danger signs (AOR = 3.18 95% CI; 2.28, 7.64). See Table 4 for more details.

Similarly, the qualitative finding also showed that Health seeking behavior of the community is low.

35 years old female FGD participants said “*I did not face any problem in my previous pregnancy because of that I didn’t go there [health center] for pregnancy follow up but in my recent pregnancy I was sick because of that I attained the clinic for examination.*

## Discussion

The finding of this research showed that mother who travels more than 30 min was more likely to drop out from the required antenatal care services than those who travel less than 30 min. This finding was consistent with the finding of the previous studies conducted in different countries. Research in Tanzania based on DHS shows that women who report distance was the barrier for health care seeking more likely underutilizing the required ANC visits [24]. Similarly, research conducted in Nigeria shows that perceived distance leads to underutilization of ANC visits [25]. Another literature review also identified distance as an important barrier for ANC services utilization [26]. This might be because some women in rural areas in developing countries spend more time on their multiple responsibilities such as care of children, collecting water or fuel, cooking, cleaning, and agricultural activity rather than on their own health. Consequently, they may not be able to travel a long distances to receive health services. In addition, findings in Ethiopia also showed that mothers who travel for less than 30 min to health facilities are more likely to receive ANC service [27]. This implies that accessibility of the health facility improved the uptakes of ANC services.

Women with low wealth index were most likely to drop out from the required antenatal care services than those from high wealth indexes. This finding was supported by the finding of previous studies conducted in Ethiopia; showed high family wealth index increases the complete utilization of ANC visits [20]. Similarly, a finding in Kenya showed that the high family wealth index increases the uptake of focused ANC service [28]. Other research conducted in Nigeria shows mothers from poor families were more likely to underutilize ANC visits [25]. This might be due to the indirect cost of antenatal care, such as transportation cost, lost agricultural production and household activities. In addition, economical status of the family also affects the uptake of the full package of antenatal care service visits [26]. This implies that improving the socio-economy of the community minimizes ANC visits drop out. The finding of the present is however different from the finding of a previous study conducted in Rwanda which showed that household wealth index was not significantly associated with ANC services utilization [14]. This might be due to, the study area. The former studies were conducted both in the urban and rural area including the capital city. In the urban setup, there are available and accessible services, which minimize indirect cost during ANC follow up.

Mothers who had poor knowledge of antenatal care services were more likely to drop out of ANC service than those who had good knowledge. This finding was supported by the finding of previous study conducted in Benin which showed lack of knowledge on the benefits of ANC leads to its underutilization [29]. This might be due to Knowledge increase the understanding of the mother on the existing ANC services, the importance of ANC services, and for whom the service was provided [26]. In addition, a high illiteracy level in the community may cause poor understandings of ANC services. This implies that improving the mother’s knowledge with regard to the importance and adequacy of antenatal care increases the uptakes of ANC services.

Mothers who had not accompanied person go with her for antenatal care visits were more likely to drop out of antenatal care services than those who have the accompanying person. This finding was consistent with the findings of the previous study conducted in Nigeria which shows that lack of an accompany person during ANC leads to underutilization of ANC visits [25]. A study in Mozambique also shows that accompanies during the antenatal care visit increases the uptake of the antenatal care visit [30]. This might be due to the culture of the rural community in Ethiopia, women are not allowed to go alone anywhere. This implies that involving males in ANC and improving women’s status in the community may increase the uptakes of the full ANC package.

Developed during danger sign during pregnancy was significantly associated with the utilization of adequate antenatal care services. Women who did not develop a danger sign during pregnancy were more likely to drop out of antenatal care visits than those who developed a danger sign. This finding is consistent with studies conducted in Ethiopia, which shows that mothers who recognize risk factors during pregnancy were more likely to utilize the services [31]. Similarly, research in South Sudan shows that women who never experience pregnancy complication, were more likely to be associated with non-utilization of ANC visits [15]. This might be due to low health care-seeking behavior, high illiteracy level, and poor accessibility of the health system in the community. This finding was different from a previous study conducted in Timor-Leste 2015 [32]. This might be due to the implementation of the 2011–2030 nation health sector strategic plan that focus on providing comprehensive, free primary care and hospital services to all and increasing the numbers of doctor’s to achieve the plan in the rural area [33]. This implies that understandings the complications of pregnancy may help improve the utilization of ANC visits.

In conclusion, the determinants of ANC visit dropout were socio-cultural, economic, accessibility of services and individual factors. In addition, the behavior of the professional, the mothers understanding of the existing services and their perception of the ANC services were factors for ANC visit dropout. Limitation this research might be prone to recall bias that may leads to false result to minimize this we limit the study in last 6 month preceded birth. Recommendation: For federal ministry of health and NGOs, Providing training on compassionate respectful care for promoting positive attitude and behavior of health workers towards clients and building trust in government health facilities from the health care provider side.

## Supplementary information

**Additional file 1.** Questionnaire that developed for collecting data for this study.

## Data Availability

All necessary data were included in the paper. Raw data is available from the corresponding author upon reasonable request.

## Abbreviations

ANC: Antenatal care
DHS: Demographic health survey
EDHS: Ethiopian demographic health survey
FANC: Focus antenatal care
SDG: Sustainable development goals
WHO: World health organization
