# Supplementary material for: Determinants of antenatal care dropout among mothers who gave birth in the last six months in BAHIR Dar ZURIA WOREDA community; mixed designs
Source: BMC Health Serv Res. 2020 Sep 10;20:846. doi: 10.1186/s12913-020-05674-9 (PMC7488008; doi:10.1186/s12913-020-05674-9)
Supplement: Supplementary file 1 — Additional file 1. Questionnaire that developed for collecting data for this study. [file 12913_2020_5674_MOESM1_ESM.docx]

| No | **SOCIO DEMOGRAPHIC CHARACTERISTICS** |  |
| --- | --- | --- |
| 101 | How old were you on last birth? | In year …………………………….. |
| 102 | Your religion? | 1. Orthodox 2. Muslim 3. Protestant 4. Others specify……….. |
| 103 | Your educational level? | 1. Unable to read and write 2. Able to read and write 3. Primary 4. Secondary school 5. Collage and above |
| 104 | Your occupation? | 1. House wife 2. Farming 3. Traders 4. Civil servant 5. Other specify …………. |
| 105 | Your marital status? | 1. Single 2. Married but Separate 3. Married and live to together 4. Divorced 5. Windowed   If the answer is not married skip to Q No 201 |
| 106 | Your husband’s educational status? | 1. Unable to read and write 2. Able to read and write 3. Primary 4. Secondary school 5. Collage and above |
| 107 | Your husband’s occupation? | 1. Farming 2. Traders 3. Civil servant 4. Other specify ………….. |
| Socio-cultural and economical characterstics | | |
| 201 | Who is the head of the house hold? | 1. Wife 2. Husband 3. Others specify …….. |
| 202 | Who is the principal income generator of the household? | 1. Husband 2. Wife 3. Both 4. Other specify …… |
| 203 | How many people live in the house hold? | ……………………… |
| 204 | How many of them under-fives children’s? | --------------------------------- |
| 205 Wealth quintile | | |
| 205.1 | How many of the following animals do this household own?  IF NONE, RECORD 0  IF 95 OR MORE, RECORD 11  IF UNKNOWN, RECORD 12   1. Milk cows, oxen or bulls? 2. Horses, donkeys, or mules? 3. Goats 4. Sheep 5. Chickens or other poultry? | 1. Milk cows, oxen or bulls? 2. Horses, donkeys, or mules? 3. Goats 4. Sheep 5. Chickens or other poultry? |
| 205.2 | Does any member of this household own any agricultural land? | 1. Yes 2. No |
| 205.3 | How many hectares of agricultural land do members of this household own?. | Hectare ………………/timad/……..  66. I don’t know ………………. |
| 205.4 | Does your household member have?  A radio?  A table?  A chair?  A bed with cotton/sponge/spring mattress?  Sofa? | Yes NO  A radio? ------------------------1 2  A table?---------------------------1 2  A chair?---------------------------1 2  A bed with cotton  /sponge/spring mattress?--------1 2  Sofa?--------------------------------1 2 |
| 205.5 | Does any members of the household own  A Watch?  A mobile phone?  Bicycle?  Motor cycle?  Animal draw cart?  Car | Yes No  A Watch 1 0  A mobile phone 1 0  Bicycle 1 0  Motor cycle 1 0  Animal draw cart 1 0  car 1 0 |
| 205.6 | Does the roof of the house made from   - Metal/corrugated iron - Sod | Yes No  -Metal /  -corrugated iron 1 2  Sod 1 2 |
| 205.7 | Does the wall of the house laminated | 1. Yes 2. No |
| 206 | Would you have a support person to go with you to receive ANC? | 1. Yes 2. No; if the answer is No skip question n^o^ 208 |
| 208 | Who is support you during receiving ANC care? | 1. Husband 2. My Child 3. My husband’s mother 4. Other specify……… |
| Previous pregnancy | | |
| 301 | How many times have you been pregnant? | 1. Once 2. 2 up to 5 3. More than 5 |
| 302 | Numbers of children ? | ……………………. |
| 303 | How many hours you travel to reach for the health services? | 1. With in 1 hour 2. 1 to 2 hour 3. More than 2 hour 4. I don’t know |
| 304 | How many Km you travel to reach ANC health care services? | By Km………………..  66. I don’t know |
| 305 | What’s your means of transportation to reach ANC services? | 1. On foot 2. On the back of the horse/mule 3. By car 4. On animal drawn cart 5. Other specify |
| 306 | Would the last pregnancy is wanted | 1. Yes 2. No |
| 307 | How many times did you receive  antenatal care during this  Pregnancy? | 1. One times 2. Two times 3. Three times 4. Four 5. Five and more times   If the answer of this question was four or more skip to question No 512 |
| 308 | If less than four, why you attained less than four ANC visit | 1. Discontinue 2. Delay registration 3. Other specifies………………… |
| 309 | If they discontinue, Why you are discontinue | 1. They don’t tell me when to come back 2. I feel health 3. Due to distances 4. Being busy 5. The services was not attracting 6. Other specify …………… |
| 310 | As part of your antenatal care during this pregnancy, were any of the following complications was develop  Vaginal bleeding?  Vaginal gush of fluid?  Severe headache?  Blurred vision?  Fever?  Abdominal pain?  Convulsion?  Other specify | Yes No  Vaginal bleeding 1 2  Vaginal gush of fluid 1 2  Severe headache 1 2  Blurred vision 1 2  Fever 1 2  Abdominal pain 1 2  Convulsion 1 2  6 Other specify ………………… |
| Mothers knowledge towards ANC | | |
| 401 | Do you think pregnant women need to go ANC clinic for antenatal check-up? | 1. Yes 2. No |
| 402 | Do you think a pregnant woman need blood pressure measurement during ANC services? | 1. Yes 2. No |
| 403 | Do you think receiving ANC during pregnancy is important? | 1. Yes 2. No |
| 404 | Do you think mothers need ANC at any time without complication? | 1. Yes 2. No |
| 405 | Do you think that ANC minimize pregnancy related complication? | 1. Yes 2. No |
| 406 | Do you think pregnant women need to start ANC services before 4 months | 1. Yes 2. No |
| 407 | Do you think pregnant woman needs at least four ANC visit for her pregnancy? | 1. Yes 2. No |
| 408 | Do you think pregnant women need iron tablet? | 1. Yes 2. No |
| Attitudes of the mother on antenatal care | | |
| 501 | When you become pregnant would you go to ANC clinic for attending Antenatal care check-up important for me and my baby | 1. Strongly agree 2. Agree 3. Neutral 4. Disagree 5. Strongly disagree |
| 502 | Would you agree, attained Antenatal follow-up is good to monitor your and your fetal health | 1. Strongly agree 2. Agree 3. Neutral 4. Disagree 5. Strongly disagree |
| 503 | would your previous experience helps you to start antenatal care booking before 4 months if you became pregnancy | 1. Strongly agree 2. Agree 3. Neutral 4. Disagree 5. Strongly disagree |
| 504 | On your previous experience, would you allow yourself to screen your blood for infection like (HIV, HBV) if you become pregnant | 1. Strongly agree 2. Agree 3. Neutral 4. Disagree 5. Strongly disagree |
| 505 | In your previous ANC experience, would you allow to check vaginal examination for the next if it’s necessary | 1. Strongly agree 2. Agree 3. Neutral 4. Disagree 5. Strongly disagree |
| 506 | In your previous ANC experience, would you modify your dietary habit as advice of professionals for your next pregnancy | 1. Strongly agree 2. Agree 3. Neutral 4. Disagree 5. Strongly disagree |
| 507 | In your previous ANC experience would you take iron and folic acid Supplementations as prescribed of professional that’s important for you and your baby | 1. Strongly agree 2. Agree 3. Neutral 4. Disagree 5. Strongly disagree |
